# Supplementary material for: Single cell transcriptomic landscape of diabetic foot ulcers
Source: Nat Commun. 2022 Jan 10;13:181. doi: 10.1038/s41467-021-27801-8 (PMC8748704; doi:10.1038/s41467-021-27801-8)
Supplement: Supplementary file 1 — Supplementary Information [file 41467_2021_27801_MOESM1_ESM.docx]

Single Cell Transcriptomic Landscape of Diabetic Foot Ulcers

Theocharidis *et al*

Supplementary Information


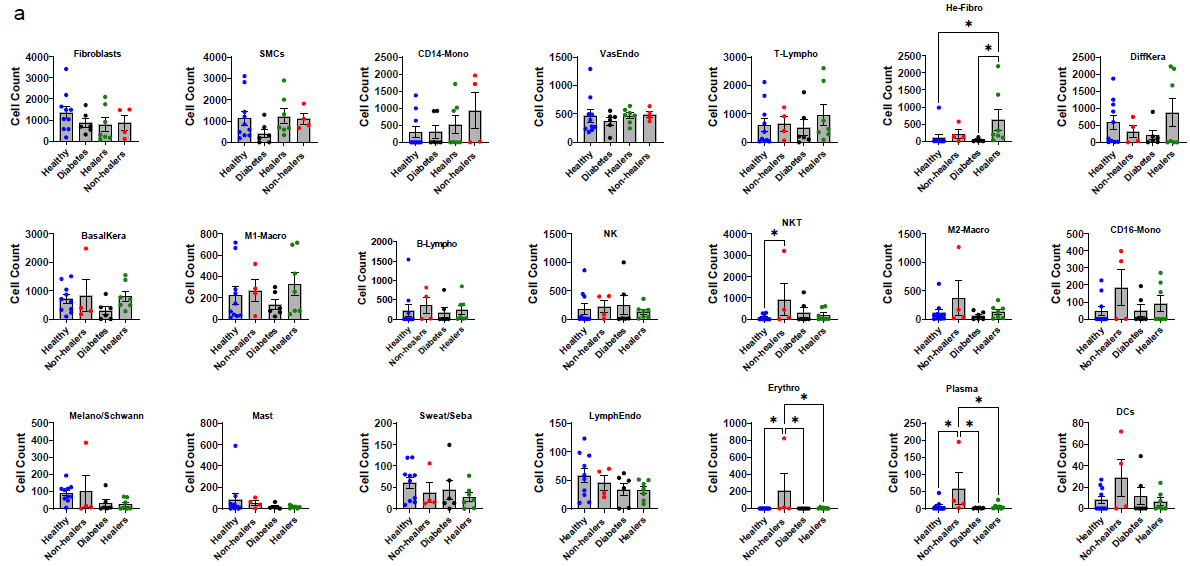


**Supplementary Figure 1. Comparative analysis of cellular abundance for each cell type across clinical groups as shown in Figure 1D**. Bar plots representing the mean and SEM values cell count for the 21 cell types in clinical groups for samples from all anatomical sites. Data represent the mean and SEM values from n = 10 Healthy, n = 6 Diabetes, n = 7 Healers, and n = 4 Non-healers subjects. Statistical analysis was performed with one-way ANOVA with Fisher’s LSD *post-hoc*. p = 0.036 for Healthy vs Healers and p = 0.034 for Diabetes vs Healers in HE-Fibro; p = 0.033 for Healthy vs Non-healers in NKT; p = 0.025 for Healthy vs Non-healers, p = 0.038 for Diabetes vs Non-healers and p = 0.037 for Healers vs Non-healers in Erythro; p = 0.019 for Healthy vs Non-healers, p = 0.017 for Diabetes vs Non-healers and p = 0.024 for Healers vs Non-healers in Plasma. These differences can be confounded with variability in the number of samples in each clinical groups as well as number of single cells captured for each sample.


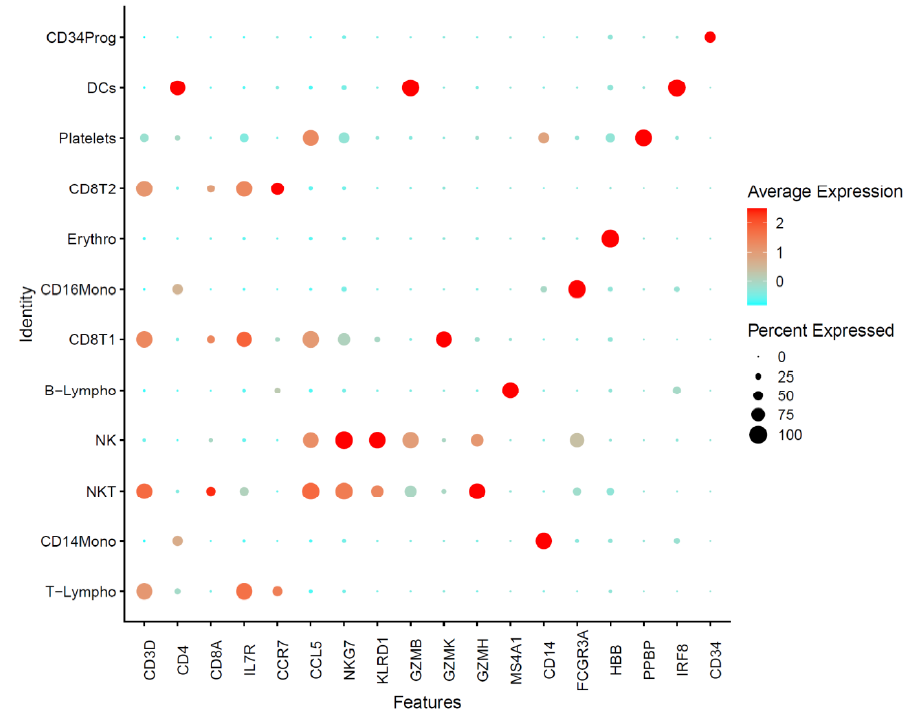


**Supplementary Figure 2. PBMC cell cluster annotation.** Cell annotation was performed based on expression of canonical marker genes. Dot plot showing expression of markers genes in 12 cell types. Size of dots indicates percentage of cells in each cluster that are expressing the marker gene; color represents averaged scaled expression levels; cyan: low, red: high.


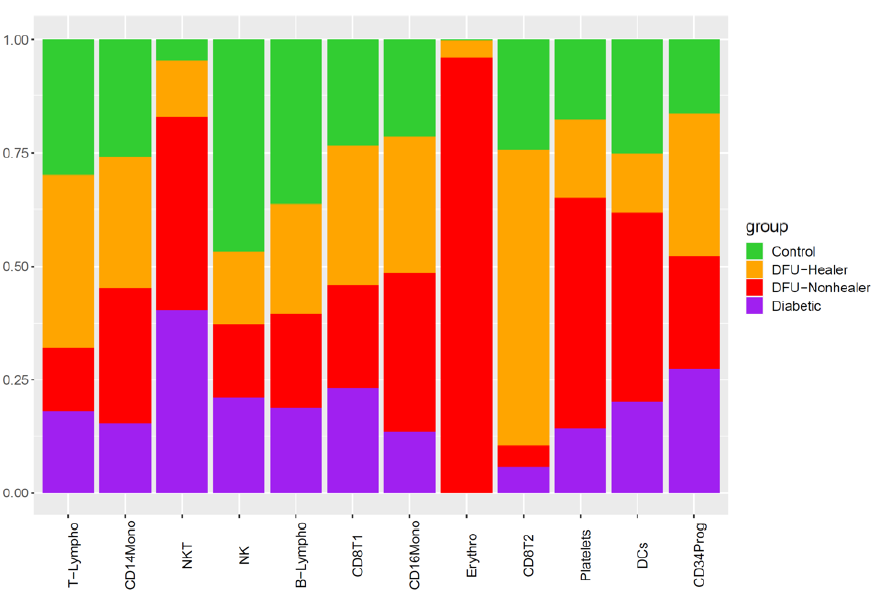


**Supplementary Figure 3. Distribution of cells from various clinical groups in PBMC cell types.** Stacked bar plots showing the proportions of different cell types across the different clinical groups (Green: Healthy controls, Orange: DFU-Healers, Red: DFU-Nonhealers, Purple: non-DFU DM).


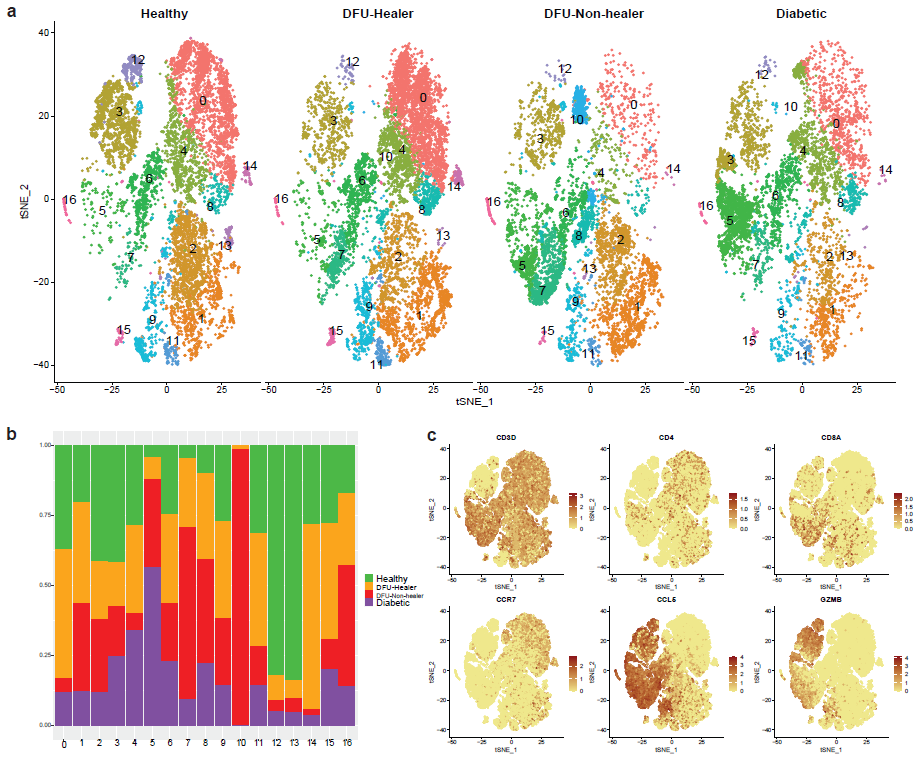


**Supplementary Figure 4.** **Comparative analysis of T, NKT, and NK cell subpopulations in different clinical groups.** **(a)** t-distributed Stochastic Neighbor Embedding (t-SNE) analysis of T-lymphocytes, Natural Killer (NK) cells and NKT cells, **(b)** The sub-cluster wise proportion of different cell subtypes across clinical groups (i.e., Green: Healthy non-DM, Orange: DFU-Healers, Red: DFU-Non-Healers, Purple: non-DFU DM).**(c)** Feature maps showing expression of gene markers for T cells (*CD3D^+^*), T-helper (*CD4^+^*), T-cytotoxic (*CD8A^+^*), Naïve/Central Memory T-cell (*CCR7^+^*), effector CD8^+^ T cells (*CCL5^+^*), and NK (*GZMB^+^ and CD3D^-^*).


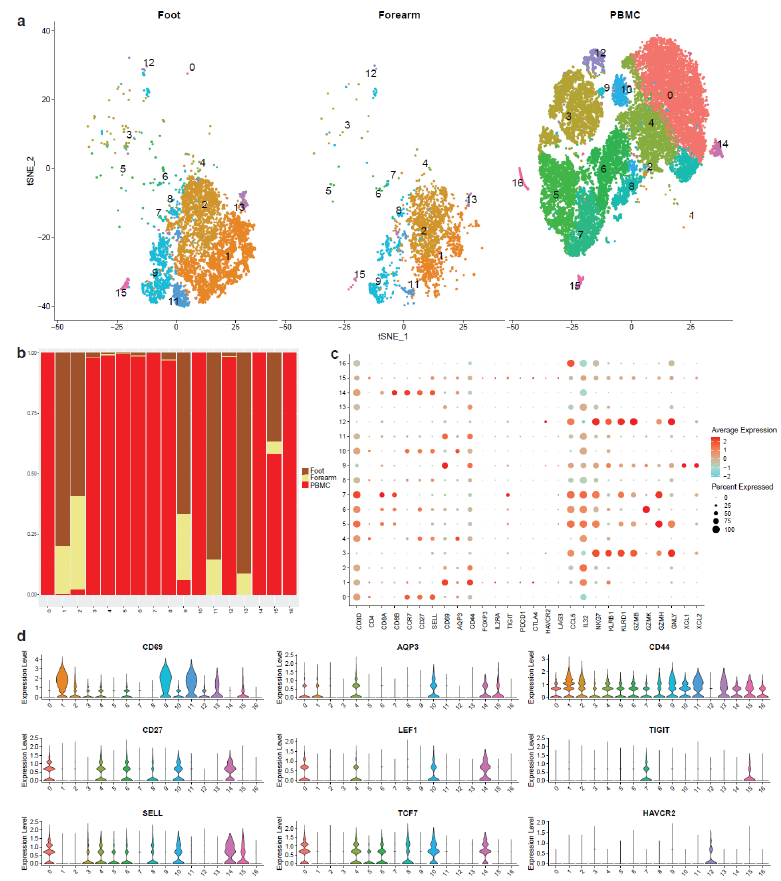


**Supplementary Figure 5. T cells and NK cells subset analysis. (a)** Split t-SNE plots based on anatomical sites, **(b)** Bar plot showing composition of each T/NK sub-cluster, **(c)** Dot plot showing expression of markers genes in different cell clusters for cell type annotation. Size of dots indicates percentage of cells in each cell cluster expressing the marker gene; color represents averaged scaled expression levels; cyan: low, red: high, **(d)** Violin plots showing expression of T cell marker genes for: activation (*CD69+, CD44+*), naive (*CD27+, SELL+, LEF1+*), memory (*AQP3+*), differentiation (*TCF7+*) and exhaustion (*TIGIT*+, *HAVCR2*+).


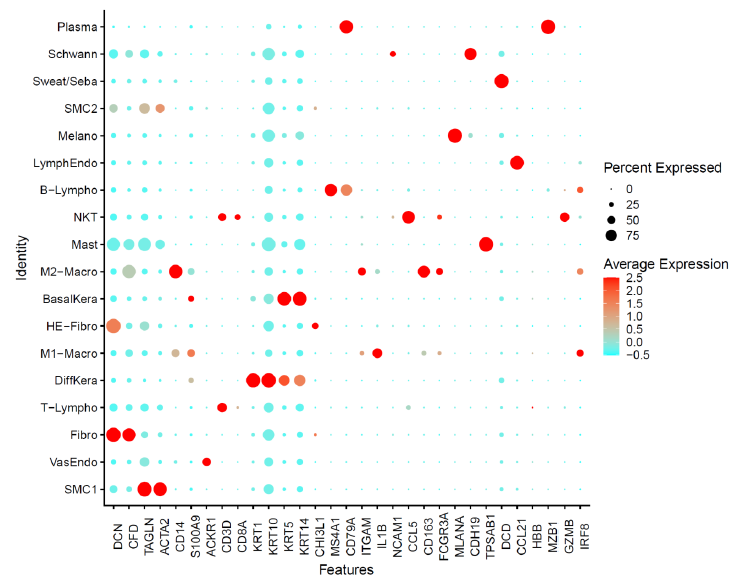


**Supplementary Figure 6. Annotation of foot clusters based on expression of marker genes.** Dot plot showing expression of markers genes in 18 clusters. Size of dots indicates percentage of cells in each cell cluster expressing the marker gene; color represents averaged scaled expression levels; cyan: low, red: high.


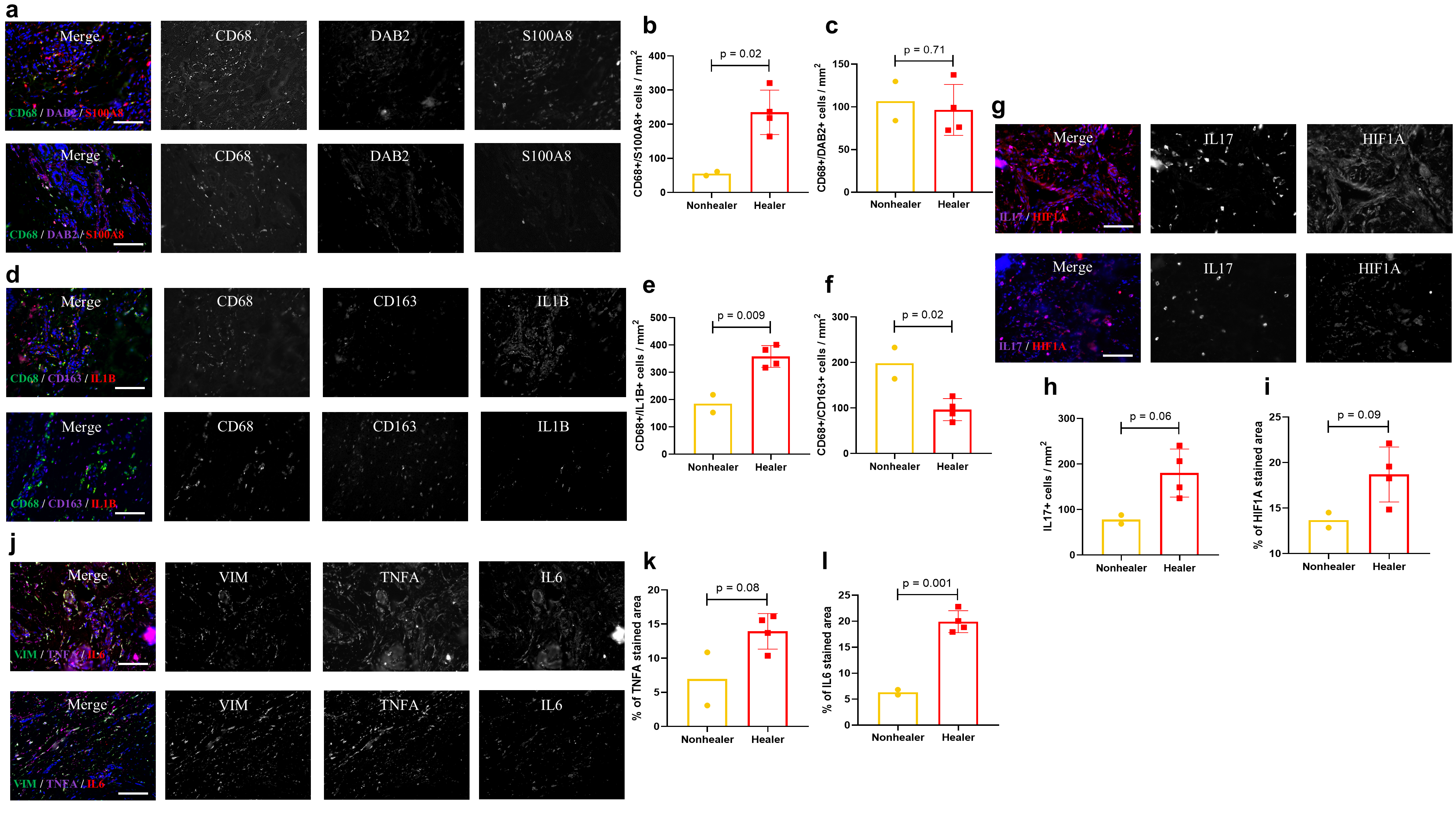


**Supplementary Figure 7. Immunofluorescent staining and quantification of macrophage polarization markers and key activated pathways and regulators.** **(a-c)** Representative images of pan-macrophage marker CD68 (green), M2 marker DAB2 (magenta) and M1 marker S100A8 (red) expression in healing (top row) and non-healing (bottom row) DFUs **(a)** and quantification of double positive cells per unit area **(b and c)**. **(d-f)** Representative images of pan-macrophage marker CD68 (green), M2 marker CD163 (magenta) and M1 marker IL1B (red) expression in healing (top row) and non-healing (bottom row) DFUs **(d)** and quantification of double positive cells per unit area **(e and f)**. **(g-i)** Representative images of IL17 (magenta) and HIF1A (red) expression in healing (top row) and non-healing (bottom row) DFUs **(g)** and quantification of positive cells per unit area **(h)** and percentage of stained area **(i)**. **(j-l)** Representative images of mesenchymal marker vimentin (VIM) (green) and inflammatory markers TNFA (magenta) and IL6 (red) expression in healing (top row) and non-healing (bottom row) DFUs **(j)** and quantification of percentage of stained area **(k and l)**. DAPI (blue) was used for nuclear counterstain. Data represent mean ± SD from n = 2 DFU-Non-Healers and n = 4 DFU-Healers. P values were calculated by two-tailed unpaired t-test. Stainings were performed three times with 2 DFU-Non-Healers and 4 DFU-Healers samples. Scale bars are 50 μm.


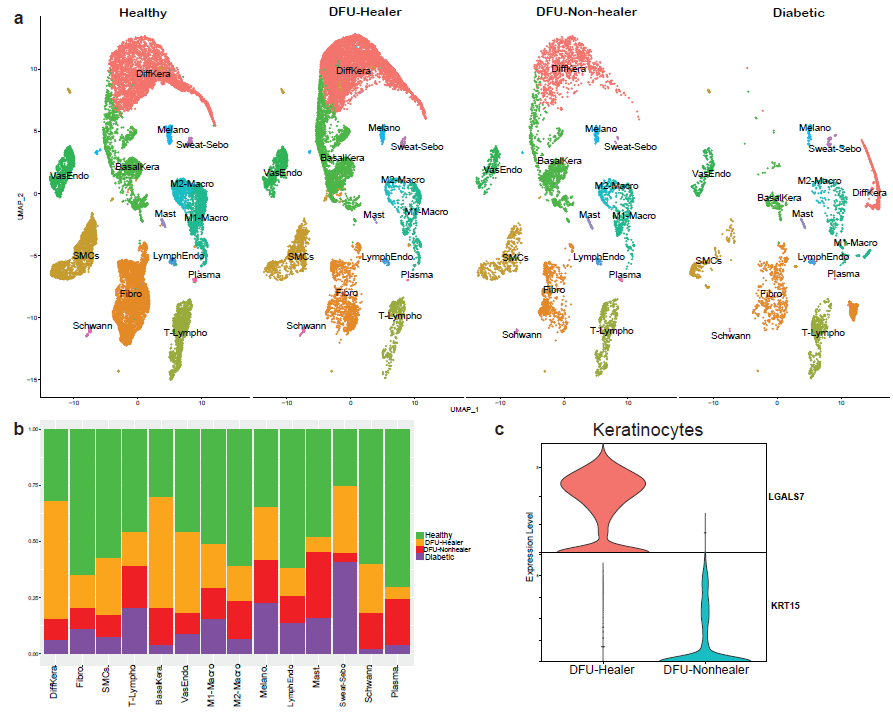


**Supplementary Figure 8. Comparative analysis of transcriptome profiles of forearm biopsies in the different clinical groups**. **(a)** UMAP dimensionality reduction embedding of forearm skin cells from DFU-Healers, DFU-Non-healers, Healthy subjects, and non-DFU DM patients. **(b)** Stacked bar plots showing the proportions of different cell types across the different clinical groups (Green: Healthy non-DM, Orange: DFU-Healers, Red: DFU-Non-Healers, Purple: non-DFU DM). **(c)** Violin plots showing expression levels of top differentially expressed gene *LGALS7* in DFU-Healers and *KRT15* in DFU-Non-healers.


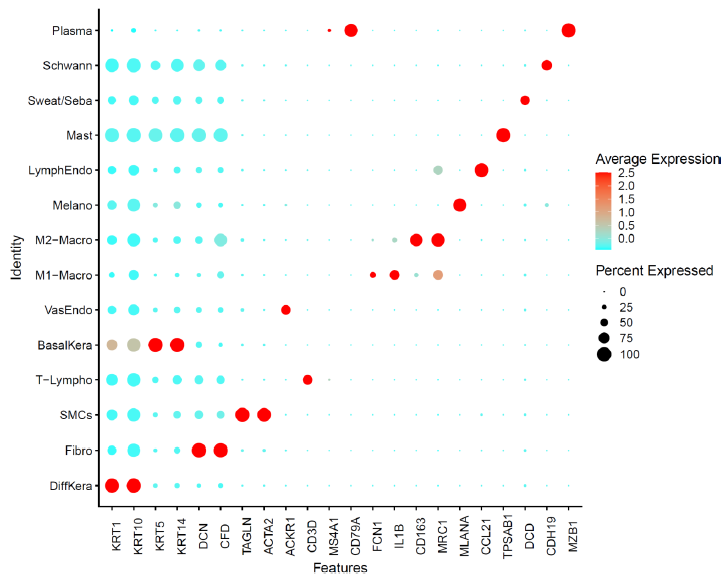


**Supplementary Figure 9. Cell type annotation of forearm cell clusters.** Dot plot showing expression of markers genes in 14 cell sub-types. Size of dots indicates percentage of cells in each cell cluster expressing the marker gene; color represents averaged scaled expression levels; cyan: low, red: high.


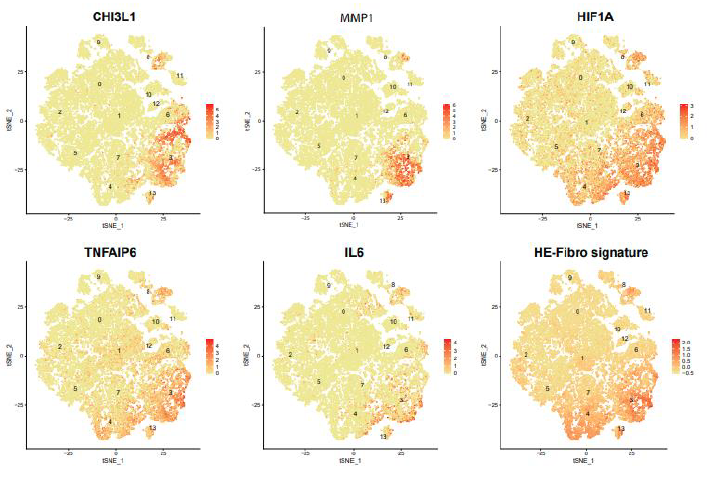


**Supplementary Figure 10. Expression of key genes for HE-Fibro**. Feature plots depicting the expression of key genes (*MMP1*, *CHI3L1*, *HIF1A*, *IL6*, *TNFAIP6*) and HE-Fibro signature (*MMP1*, *MMP3*, *IL6*, *CHI3L1*, *ASPN*, *POSTN*, *PLA2G2A*) across healing associated fibroblast sub-clusters.


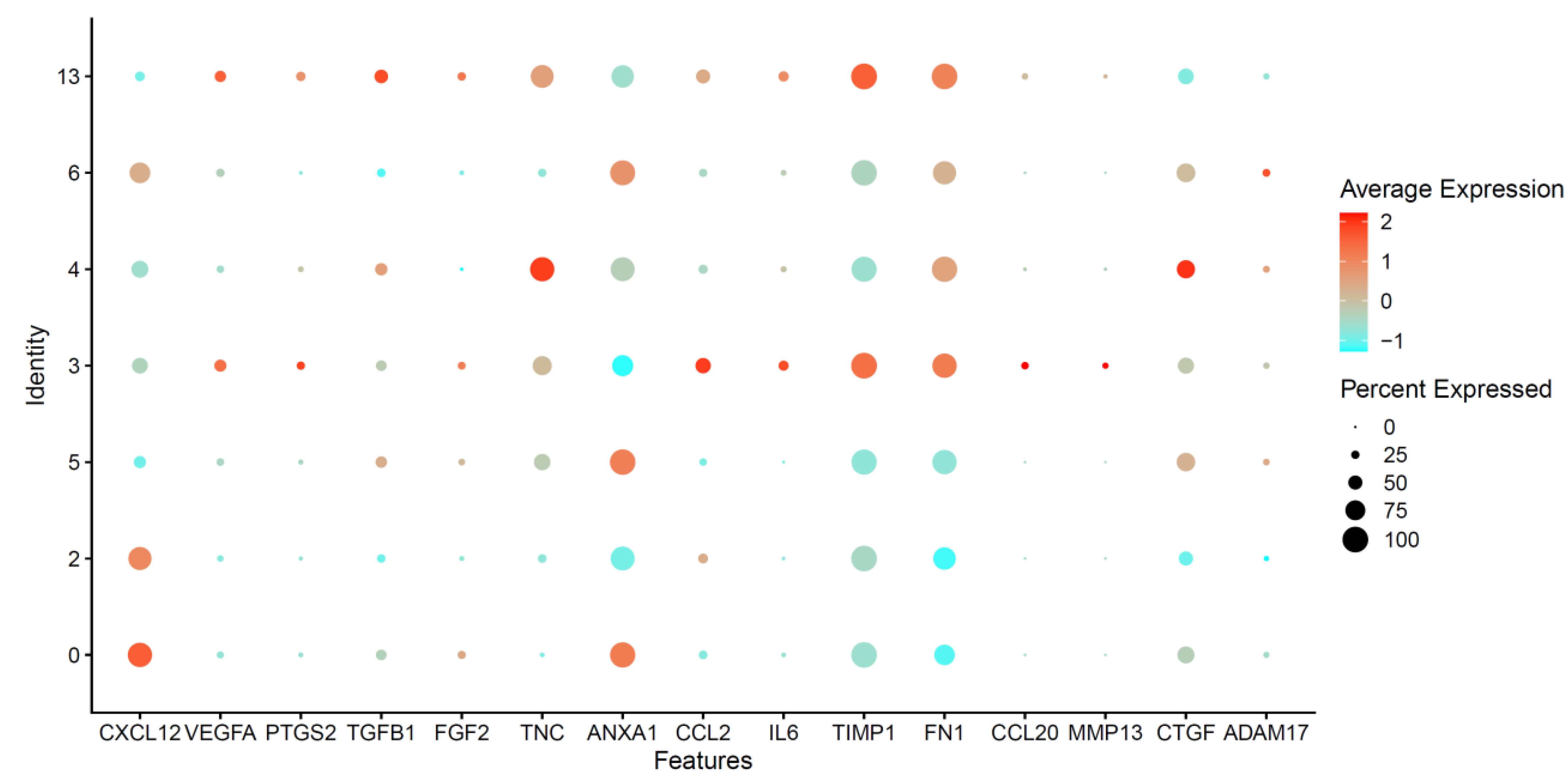


**Supplementary Figure 11. Dot Plot shows the expression of some of the top ligands based on Pearson correlation expressed by the healer-enriched fibroblasts**. X-axis shows the genes and Y-axis the cluster number. DFU-Healer enriched clusters (3, 4, 6, 13) show higher expression of *FN1, MMP13, TIMP1, CCL2,* IL6 and *CCL20* relative to DFU-Nonhealer specific fibroblast clusters (0, 2, 5).


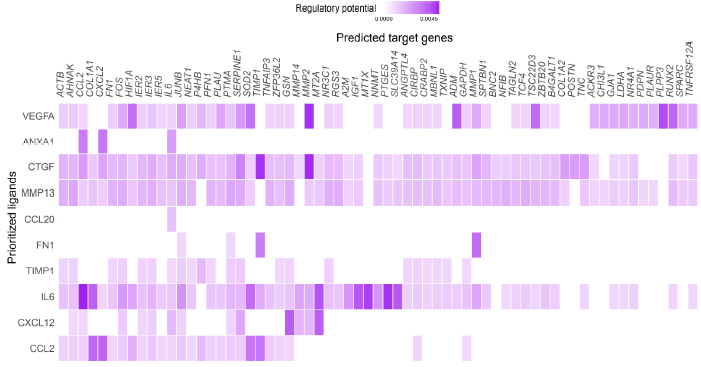


**Supplementary Figure 12. Heatmap of ligand-target interactions between the top ligands expressed by HE-Fibro cluster 3, and the differentially expressed genes of the ‘healer’ fibroblasts.** Darker purple boxes indicate a higher regulatory potential, for which there is a stronger established association between the given ligand (rows) regulating the expression of a given target gene (columns). This figure has a lower regulatory potential cutoff, to both show additional ligand-target interactions, and weaker ligand-target interactions for previously included ligand-target pairs.


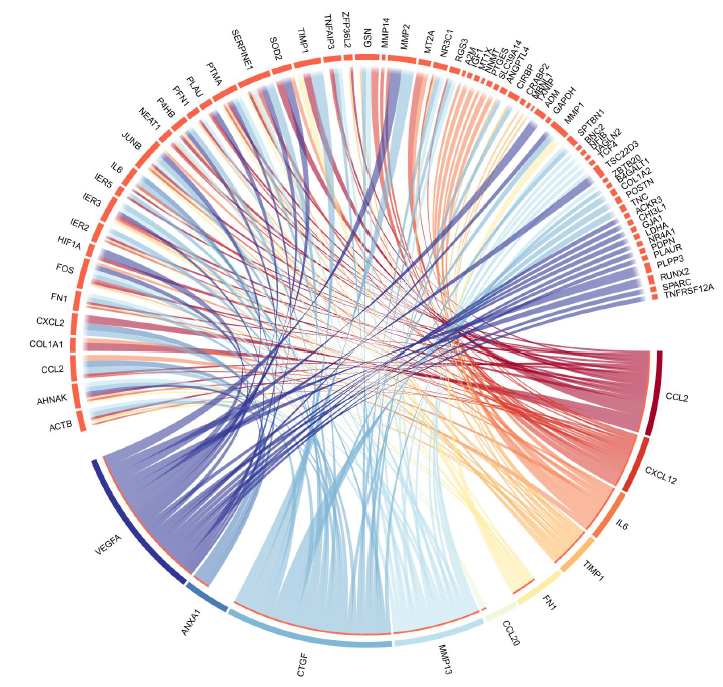


**Supplementary Figure 13. Circos plot displaying the association between ligands expressed in HE-Fibroblast subcluster 3 (bottom semi-circle) with the differentially expressed genes in the remaining HE-Fibroblast clusters.** Chords connecting ligands with the genes they are predicted to regulate. This figure has a lower cutoff for ligand-target interactions, displaying more associations between the ligands expressed by HE-Fibroblast cluster 3 and the HE-Fibroblast marker genes.


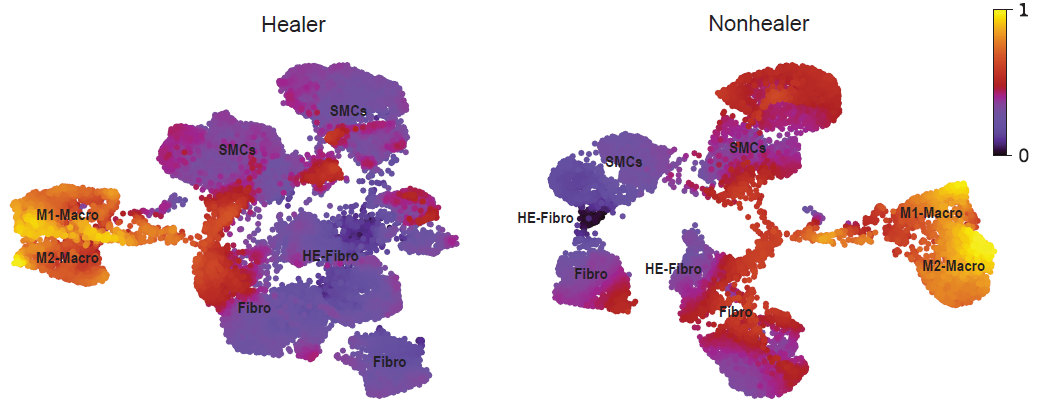


**Supplementary Figure 14. Cellular latent time estimation for DFU-Healers and DFU-Non-healers based on transcriptional dynamics.** **(Left panel)** Latent time plot for DFU-Healer group in M1 macrophages (M1-Macro), M2-Macro, healing enriched fibroblasts (HE-Fibro), fibroblasts (Fibro), and smooth muscle cells (SMCs). Latent time estimates a cell’s internal clock; 0 represents a cell early in the biological process, and 1 represents a cell late in a biological process. The analysis revealed that HE-Fibro, SMCs, Fibro in DFU-Healers had latent times < 0.5 indicating that these cells were at an early stage of differentiation. **(Right Panel)** Latent time plot for DFU-Non-healer group in M1-Macro, M2-Macro, HE-Fibro, Fibro, and SMCs. Overall latent time for HE-Fibro, SMCs, Fibro and Macrophages was >0.5 in DFU-Non-Healers indicating that these cells were more likely in a differentiated stage.


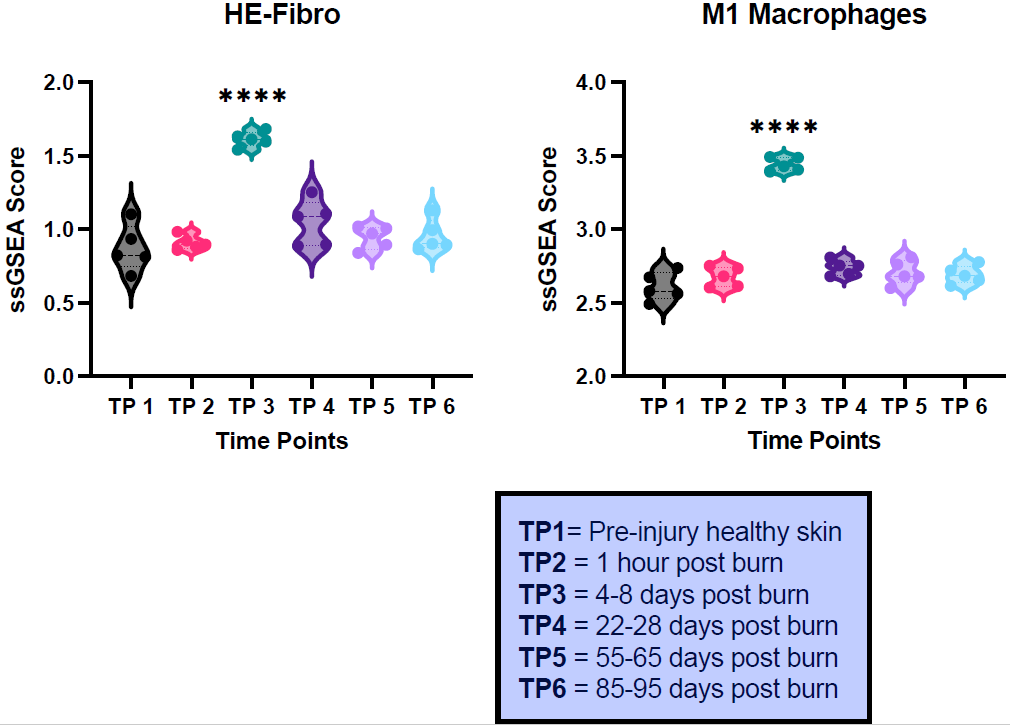


**Supplementary Figure 15. HE-Fibro and M1-Macro signatures enrichment analysis in temporal acute wound healing data using Single Sample Gene Set Enrichment Analysis (ssGSEA) analysis.** **(Left panel)** Violin plots showing distribution of ssGSEA score for the HE-Fibro signature for each timepoint during acute burn wound healing. One way mixed-effects ANOVA test with Bonferroni corrected p-value was conducted for comparing ssGSEA score across multiple time points. Timepoint 3 (TP3 = 4-8 days post-wounding) in comparison to all other timepoints (TP1, TP2, TP4, TP5 and TP6) had significantly higher ssGSEA scores (n = 5; **** p-value < 0.0001). **(Right panel)** Violin plots showing distribution of ssGSEA score M1-Macro signature for each timepoint during burn wound healing. TP3 in comparison to all other timepoints had significantly higher ssGSEA scores (n = 5; **** p-value < 0.0001). Acute burn wound data were downloaded from ArrayExpress, accession ID [E-MTAB-1323](https://www.ebi.ac.uk/arrayexpress/experiments/E-MTAB-1323/).


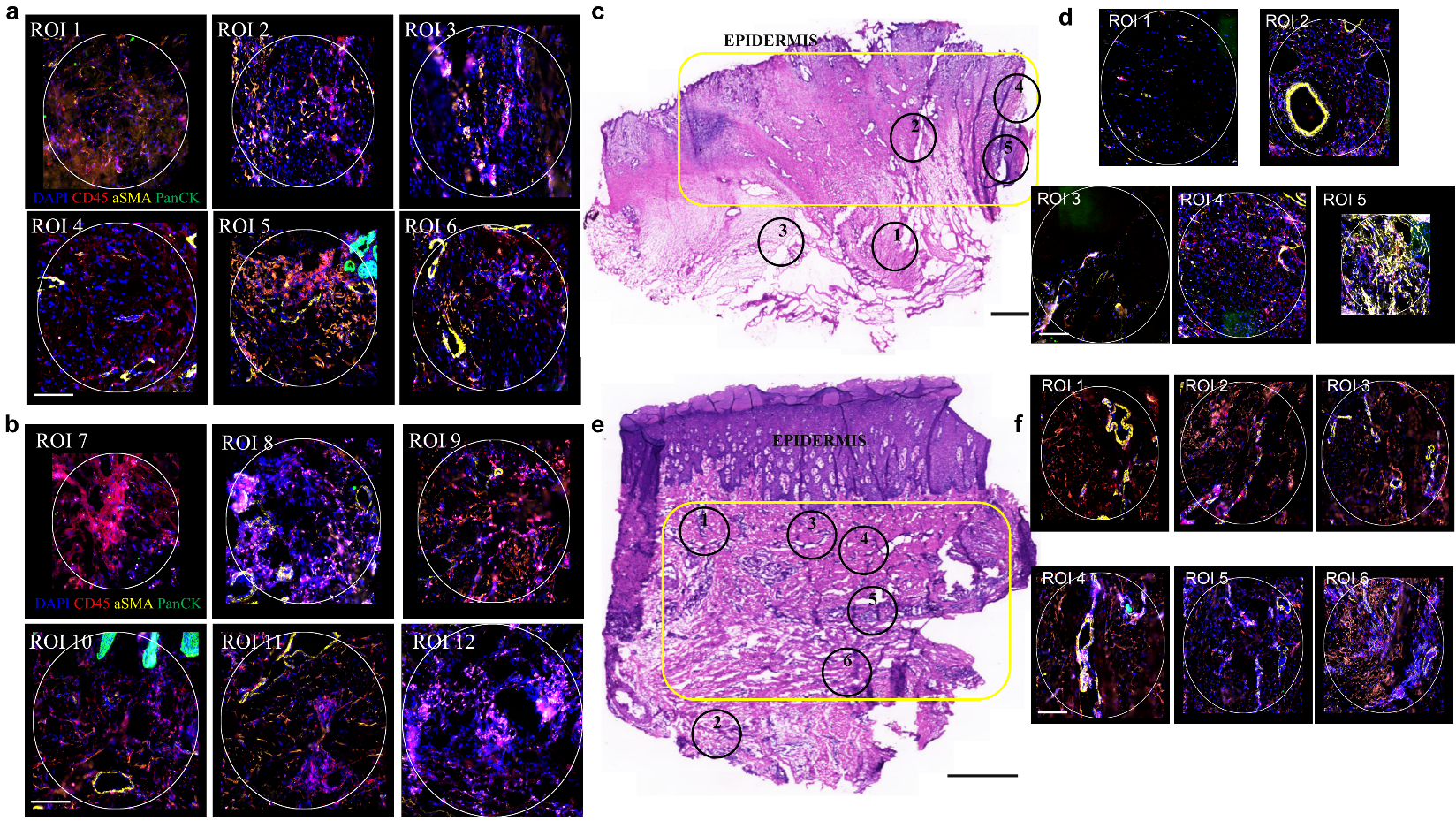


**Supplementary Figure 16. Regions of interest (ROIs) selection** (**a, b**) Micrographs presenting the ROIs selected for spatial transcriptomics analysis from a non-healing (**a**) and healing (**b**) DFU labeled by immunofluorescence as shown of Figure 6. (**c,e**) Representative H&E stained sections from a non-healing (**c**) and a healing (**e**) DFU. Yellow box demarcates the ulcer area and numbered circles the ROIs selected for sequencing. (**d,f**) The immunofluorescence staining of ROIs for markers CD45 (red), aSMA (yellow) and panCytokeratin (green). DAPI was used for nuclear counterstain. Staining was performed once with two biologically independent patient samples per group. Scale bars are 100 μm in (**a,b,d,f**) and 1 mm in (**c,e**).


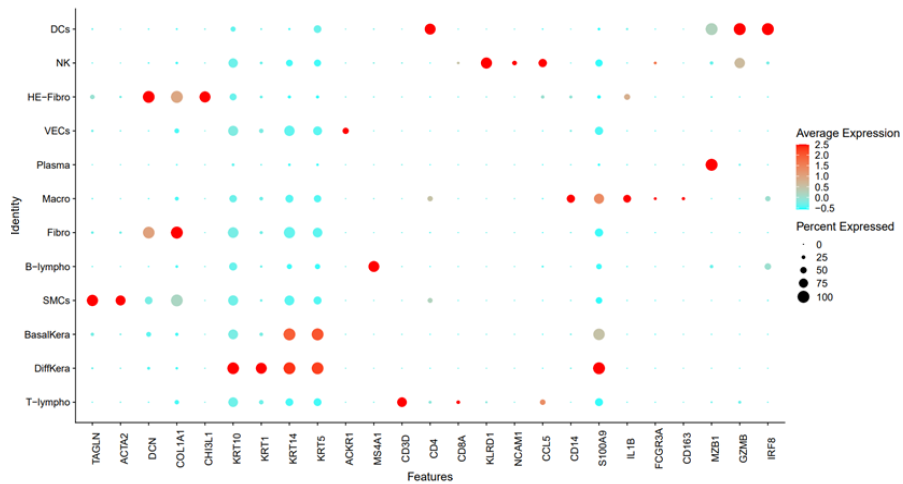


**Supplementary Figure 17. Cell type annotation of pressure sore cell clusters.** scRNAseq analysis was performed on skin specimens of the same patient from three different sites: wound bed, wound edge, and non-wound excess skin from a pressure sore excision. Dot plot showing expression of markers genes in the identified 12 cell types. X-axis shows the genes and Y-axis the cell type. Size of dots indicates percentage of cells in each cell cluster expressing the marker gene; color represents averaged scaled expression levels; cyan: low, red: high.


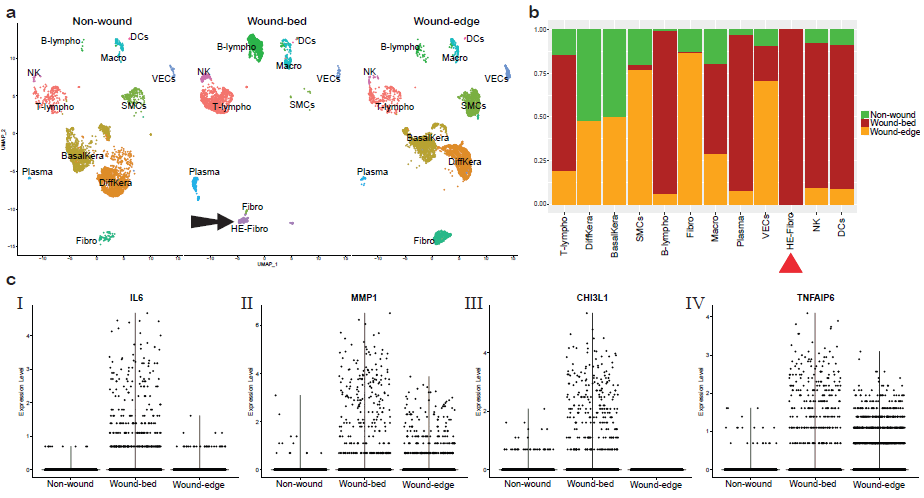


**Supplementary Figure 18. Healing Enriched Fibroblasts (HE-Fibro) are exclusively localized to the wound bed. (a)** UMAP showing distribution of different cell types in three samples from the non-wound site, wound bed and wound edge from an ischial pressure sore of one patient. Black arrow indicates the HE-Fibro cell cluster. **(b)** Bar plot showing relative proportions of cells from the 3 samples in the different cell types. Red triangle indicates the HE-Fibro cell cluster exclusively present in the wound bed. **(c)** Expression levels of (I) *IL6*, (II) *MMP1*, (III) *CHI3L1*, (IV) *TNFAIP6*, which were found to be overexpressed by the HE-Fibro cells in our study, showed higher expression in the wound bed as compared to the other sites.


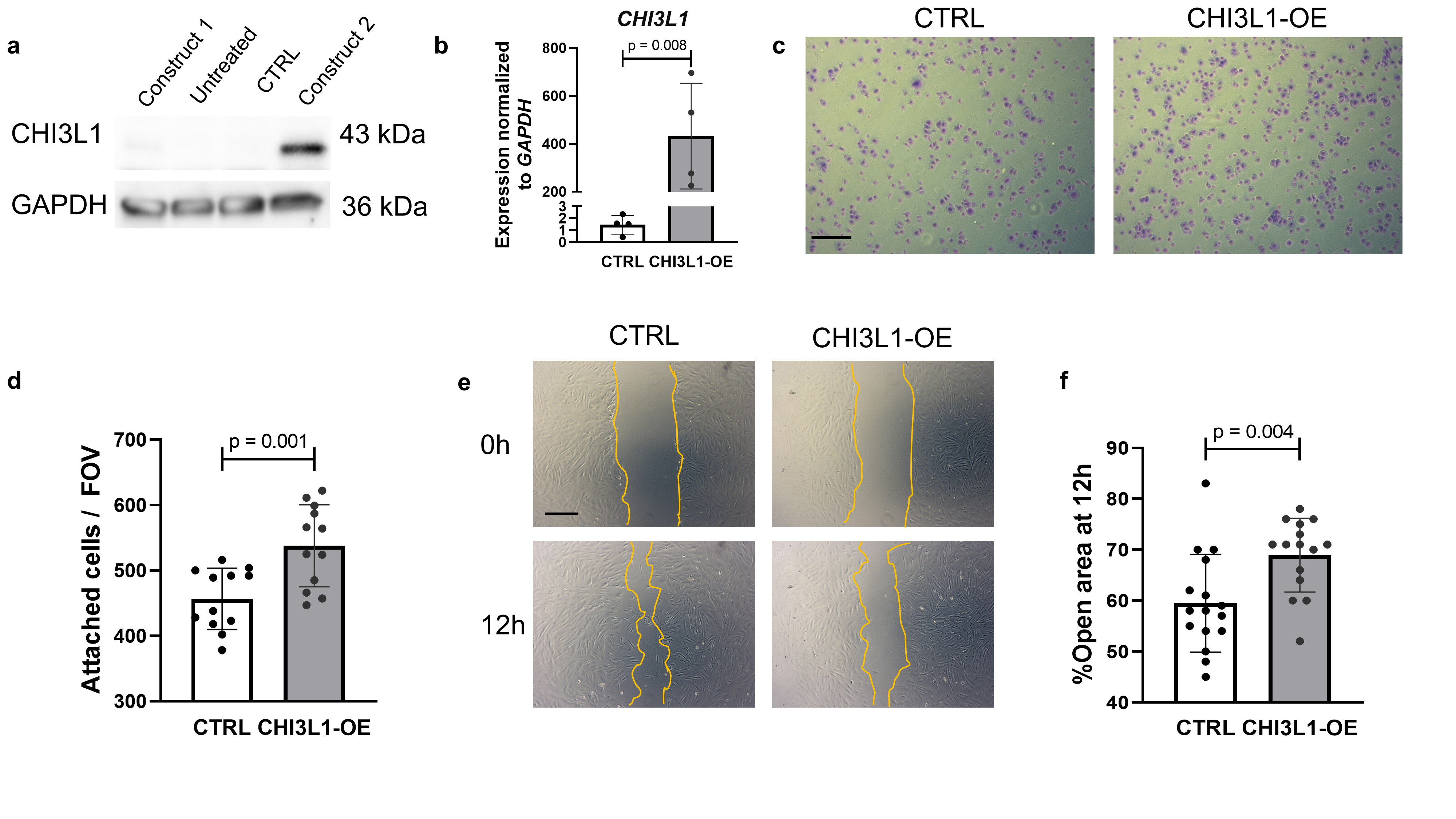


**Supplementary Figure 19. CHI3L1 overexpression in dermal fibroblasts. (a)** Western blot analysis of two constructs (1 and 2) overexpressing CHI3L1, untreated cells and control (CTRL) construct expressing cells. GAPDH was used as a loading control. **(b)** RT-qPCR of *CHI3L1* for CTRL and Construct 2 (CHI3L1-OE) expressing cells. **(c,d)** Representative images **(c)** and quantitation **(d)** of crystal violet stained cells that attached after 1 hour on fibronectin coated wells. **(e,f)** Representative images **(e)** and quantitation **(f)** of scratch wounds at 0 and 12 hours. Data represent mean ± SD of n = 4 biologically independent experiments for **(b),** n = 12 observations from three biologically independent experiments for **(d)** and n = 16 observations for CTRL and n = 15 observations for CHI3L1-OE from three biologically independent experiments for **(f)**. P values were calculated by two-tailed unpaired t-test. The experiments on **(a)**, **(c)** and **(e)** were repeated three times. Scale bars in **(c,e)** are 100 μm. FOV: Field Of View.

**Supplementary table 1. Distribution of different cell types across anatomical locations.** Table showing average percentage of each cell type in three sample types, namely foot, forearm and PBMCs. The cell type percentage were calculated as percent of total cells of the specific cell type from the three anatomical sites of collection. Standard error (SE) was calculated as the square root of the standard deviation between the number of cells present in each patient sample.

| **Cell type** | **Average % of cells ± SE** | |  |
| --- | --- | --- | --- |
|  | **Foot** | **Forearm** | **PBMCs** |
| Fibro | 73.54 ± 0.50 | 26.46 ± 0.54 |  |
| SMCs | 86.65 ± 0.61 | 13.35 ± 0.22 |  |
| CD14-Mono | 1.53 ± 0.04 |  | 98.47 ± 1.06 |
| VasEndo | 82.37 ± 0.33 | 17.63 ± 0.29 |  |
| T-lympho | 31.27 ± 0.27 | 12.42 ± 0.35 | 56.31 ± 1.21 |
| DiffKera | 17.26 ± 0.69 | 73.28 ± 1.73 |  |
| BasalKera | 71.35 ± 0.48 | 28.65 ± 0.72 |  |
| HE-fibro | 99.94 ± 1.58 | 0.06 ± 0.00 |  |
| B-Lympho | 15.51 ± 0.27 |  | 84.49 ± 1.63 |
| M1-Macro | 55.23 ± 0.55 | 36.75 ± 0.65 | 8.02 ± 0.11 |
| NK | 12.08 ± 0.15 | 2.88 ± 0.06 | 85.04 ± 1.11 |
| NKT | 3.78 ± 0.04 | 2.01 ± 0.07 | 94.21 ± 3.62 |
| M2-Macro | 71.28 ± 1.27 | 28.62 ± 1.09 | 0.10 ± 0.01 |
| CD16-Mono | 0.28 ± 0.01 | 0.09 ± 0.00 | 99.63 ± 1.11 |
| Melano/Schwann | 58.01 ± 0.76 | 41.99 ± 0.81 |  |
| Mast | 82.51 ± 1.49 | 17.34 ± 0.74 | 0.16 ± 0.01 |
| Sweat/Seba | 85.96 ± 0.74 | 14.04 ± 0.41 |  |
| LymphEndo | 75.42 ± 0.39 | 24.58 ± 0.53 |  |
| Erythro | 1.72 ± 0.06 |  | 98.28 ± 5.82 |
| Plasma | 66.01 ± 1.89 | 19.38 ± 1.18 | 14.61 ± 0.46 |
| DCs | 1.92 ± 0.04 | 4.49 ± 0.38 | 93.59 ± 1.99 |

**Supplementary Table 2: Clinical characteristics of patients across groups.** * Healthy Controls vs DM without DFU, DFU-Healers and DFU-Non-Healers; p<0.05, one- way ANOVA with Fisher’s LSD *post-hoc*.

|  | **Healthy Controls** | **DM without DFU** | **DFU-Healers** | **DFU-Non-Healers** |
| --- | --- | --- | --- | --- |
| Number (males) | 10 (4) | 6 (2) | 7 (2) | 4 (2) |
| Age (years) | 59 ± 13 | 64 ±12 | 58 ± 18 | 50 ± 11 |
| Diabetes duration (years) | -- | 10 ± 6 | 13 ± 6 | 19 ± 22 |
| BMI (kg/m^2^) | 28.3 ± 3.8 | 32.1 ± 8.8 | 35.6 ± 6.08 | 43.1 ± 19.5 |
| HbA1c ***** (%) | 5.5 ± 0.7 | 8.4 ± 1.7 | 9.7 ± 3.6 | 7.8 ± 1.1 |
| Creatinine (mg/dL) | 0.9 ± 0.2 | 1.1 ± 0.4 | 1.1 ± 0.5 | 1.9 ± 1.4 |
| Blood Urea Nitrogen (mg/dL) | 16 ± 4 | 27 ± 17 | 22 ± 14 | 34 ± 23 |
| Cholesterol, Total (mg/dL) | 180 ± 29 | 145 ± 34 | 168 ± 64 | 137 ± 18 |
| Triglycerides (mg/dL) | 115 ± 42 | 151 ± 80 | 123 ± 72 | 272 ± 167 |
| LDL Cholesterol (mg/dL) | 88 ± 33 | 65 ± 19 | 76 ± 52 | 64 ± 10 |
| Wound Surface Area (cm²) | -- | -- | 1.0 (0.2 : 3.8) | 1.2 (0.3 : 22.8) |

*:
