# Supplementary material for: Single cell transcriptomic landscape of diabetic foot ulcers
Source: Nat Commun. 2022 Jan 10;13:181. doi: 10.1038/s41467-021-27801-8 (PMC8748704; doi:10.1038/s41467-021-27801-8)
Supplement: Supplementary file 3 — Description of Additional Supplementary Files [file 41467_2021_27801_MOESM3_ESM.docx]

File Name: Supplementary Data 1

Description: Table showing the top ten marker genes for each cell type cluster with average fold-change and adjusted p value. The fold change was calculated by comparing the expression profiles of the target cell type cluster with other cell types.

File Name: Supplementary Data 2

Description: Table showing top ten marker genes for each cell type from the foot samples with average fold-change and adjusted p value. The fold change was calculated by comparing the expression profiles of target cell type with rest of the cell types.

File Name: Supplementary Data 3

Description: Table showing top ten marker genes overexpressed in the fibroblast sub-clusters, for each sub-cluster with average fold-change (FC) and adjusted p value. The fold change was calculated by comparing the expression profiles of target cluster with rest of the cluster.

File Name: Supplementary Data 4

Description: Detailed clinical information of all human subjects
